# SU4312 Represses Glioma Progression by Inhibiting YAP and Inducing Sensitization to the Effect of Temozolomide

**DOI:** 10.3390/jcm11164765

**Published:** 2022-08-16

**Authors:** Xu Wang, Yi Zhou, Yan Wang, Xiang Wang, Yu Zhang, Yufei Mao, Long Zhang, Ji Qi, Yining Zhang, Feng Lyu, Linbo Gu, Rutong Yu, Xiuping Zhou

**Affiliations:** 1 Institute of Nervous System Diseases, Xuzhou Medical University, 84 West Huaihai Road, Xuzhou 221002, China; 2 Department of Neurosurgery, The Affiliated Hospital of Xuzhou Medical University, 99 West Huaihai Road, Xuzhou 221002, China; 3 The Graduate School, Xuzhou Medical University, Xuzhou 221004, China

**Keywords:** glioma, SU4312, YAP, temozolomide, CCL2

## Abstract

SU4312, initially designed as a multi-target tyrosine kinase inhibitor, is consequently reported to inhibit tumor angiogenesis by blocking VEGFR. However, although SU4312 can penetrate the brain–blood barrier, its potential to inhibit glioma growth is unknown. In this study, we report that SU4312 inhibited glioma cell proliferation and down-regulated yes-associated protein (YAP), the key effector of the hippo pathway. The exogenous over-expression of YAP partially restored the inhibitory effect of SU4312 on glioma progression. Interestingly, SU4312 sensitized the antitumor effect of temozolomide, both in vitro and in vivo. Moreover, SU4312 decreased the M2tumor-associated macrophages and enhanced anti-tumor immunity by down-regulating the YAP-CCL2 axis. In conclusion, our results suggest that SU4312 represses glioma progression by down-regulating YAP transcription and consequently CCL2 secretion. SU4312 may be synergistic with temozolomide for glioma treatment.

## 1. Introduction

Malignant glioma is the most common type of primary brain tumor with poor overall prognosis, and high rates of mortality and disability [1,2]. The median survival time for glioblastoma (GBM), the most malignant type of glioma, is only 14 months [1,2]. Glioma treatment includes maximal surgical resection, radiotherapy, chemotherapy and immunotherapy, however, the therapeutic effects of these are not satisfactory [3,4]. Therefore, it is urgent to explore new chemotherapeutic agents or targeted agents to treat glioma.

Receptor tyrosine kinase inhibitors are one classical type of small-molecule anti-tumor drug [5,6,7,8]. 3-[4-(dimethylamino)benzylidenyl]indolin-2-one (SU4312), initially designed as a multi-target receptor tyrosine kinase inhibitor [9], is found to inhibit tumor angiogenesis by blocking VEGFR [10]. SU4312 competes with ATP to bind to VEGFR-2 and is able to completely block VEGF signaling in a non-competitive manner [9]. In addition, SU4312 is reported to ameliorate Alzheimer’s disease and Parkinson’s disease by inhibiting angiogenesis [11] and suppressing the activity of monoamine oxidase-B, respectively [12]. Meanwhile, Cui et al. showed that SU4312 exhibits neuroprotective effects independent of its anti-angiogenic mechanism and could penetrate the blood–brain barrier [13]. Furthermore, SU4312 is also found to directly reduce the proliferation of multiple myeloma and leukemia tumor cells in vitro [13,14]. Since SU4312 is able to pass the blood–brain barrier, its anti-glioma properties are worth exploring.

Yes-associated protein (YAP) is a central effector of the hippo kinase cascade and usually plays an important role in the cancer-promoting process of most human tumors [15,16,17,18]. When the hippo kinase cascade is turned off, YAP enters the nucleus and interacts with the TEA structural domain (TEAD) family of transcription factors, leading to downstream target gene expression and tumor growth [19,20]. Many studies have clarified that YAP is highly expressed in a variety of human solid tumors and is proved to be a carcinogen [21,22,23]. Through a series of studies, we have found that the expression level of YAP in gliomas is up-regulated and correlates with the pathological grade of gliomas [24,25]. YAP intervention inhibits the growth, invasion and migration of gliomas [25,26,27]. In short, YAP is strongly associated with the malignant progression of gliomas; therefore, targeting YAP may contribute to the treatment of gliomas [27,28,29]. As a tyrosine kinase inhibitor, pazopanib inhibits the expression of YAP and its target genes [30]. SU4312 has similar pharmacological effects of pazopanib. Whether it can inhibit the expression and function of YAP needs further study.

In a retrospective cohort study, the application of temozolomide (TMZ), the first-line drug for glioma treatment, prolongs the median survival time of glioma patients [31,32]. However, TMZ treatment induces drug resistance and loses effectiveness in suppressing gliomas [33]. Therefore, how to sensitize the efficacy of TMZ has become a new research hotspot. Apatinib has been reported to inhibit cell growth and metastasis to enhance the antitumor activity of TMZ in gliomas [34]. Similarly, the combination of sunitinib and TMZ was more effective than TMZ alone for glioma treatment [35]. Therefore, as a receptor tyrosine kinase inhibitor, SU4312 deserves further study on whether and how it induces sensitization to the effect of TMZ.

In the present study, we investigated the effect of SU4312 on glioma progression, both in vitro and in vivo. We found that SU4312 hindered glioma progression by down-regulating YAP and its target gene CCL2. Furthermore, SU4312 and TMZ showed a synergistic inhibition effect on glioma progression.

## 2. Materials and Methods

### 2.1. Cell Lines, Antibodies, Reagents and Plasmids

The glioma cell lines (U251, U87, U373, LN229, GL261) were purchased from Shanghai Cell bank, Type Culture Collection Committee, Chinese Academy of Sciences. Normal human astrocyte (NHA) was purchased from ScienCell. GBM1 and GBM2 are primary glioma cells established by our laboratory [36,37,38]. Normal human astrocyte was cultured in astrocyte medium (ScienCell; Cat No.1801), while glioma cells were cultured in Dulbecco’s modified Eagle medium (DMEM) and supplemented with 10% FBS. The above cell lines were grown in a humidified incubator at 37 °C with 5% CO_2_. SU4312 and TMZ were purchased from Topscience (Shanghai, China). Primary antibodies of anti-YAP (for western blot), anti-AXL, anti-p-H2AX, anti-Iba-1, anti-Arg-1 and anti-GAPDH were obtained from Cell Signalling Techniques. Anti-YAP (for immunofluorescence) was purchased from Sigma-Aldrich. Anti-CYR61 and anti-Ki67 were purchased from Santa Cruz Biotechnology. Control and the YAP wild-type plasmids were kindly gifted by Prof. Hongbin Ji at the Institute of Biochemistry and Cell Biology, Shanghai Institute of Biological Sciences, Chinese Academy of Sciences [39].

### 2.2. Establishment of Stable YAP Over-Expression Cells

U251 and GBM1 cell lines over-expressing YAP were constructed by lentiviral transfection as described previously [36].

### 2.3. Cell Viability Assay and Colony Formation Assay

Glioma cell lines were inoculated with 5000 cells per well in 96-well plates and incubated overnight. Cells were then treated with vehicle (0.1% dimethyl sulfoxide, DMSO) or with various doses of SU4312 for 24 h. CCK8 (10 μL) reagent was then added to each well and incubated for 2 h. The adsorption rate was measured at a wavelength of 450 nm. For colony formation assay, 1000 cells were seeded into six-well plates and treated with SU4312 (10 μM) or TMZ (300 μM) for 48 h. After having been cultured for two weeks, the cells were fixed with 4% paraformaldehyde and stained with 0.05% crystal violet. The plates were dried at room temperature and the number of colonies was counted.

### 2.4. EdU Incorporation Assay

Cell proliferation was detected using the Cell-Light™ EdU Cell Proliferation Assay Kit (Ruibo Biotechnology, Guangzhou, China) according to the manufacturer’s instructions. U251 and GBM1 cells were treated with vehicle or with different concentrations (10 μM and 20 μM) of SU4312 for 24 h. The cells were incubated with 50 μM EdU for 2 h, fixed in 4% paraformaldehyde for 15 min, and then treated with 0.5% Triton X-100 for 20 min. Thereafter, cells were incubated in 1 × Apollo^®^ reaction mixture for 30 min, followed by DAPI staining for 15 min. After being washed with phosphate buffered saline (PBS) three times, the cells were observed with an inverted fluorescent microscope (Olympus, Tokyo, Japan) and photographed.

### 2.5. Cell Migration Assay

When the U251 and GBM1 cells reached 90% confluence, they were scratched with the tip of a plastic pipette, washed with PBS and then cultured with serum-free medium containing vehicle or SU4312 (10 μM). Twenty-four or forty-eight hours later, three random fields at the scratches were photographed. The number of migrated cells in the scratches was calculated for statistical analysis.

### 2.6. Transwell Invasion Assay

The Transwell system was used to determine cell invasiveness. A final concentration of Matrigel (1 mg/mL, 50 μL) was added to the bottom of the upper chamber. After the matrigel was dried, cells in a serum-free medium containing vehicle or SU4312 (10 μM) were added to the chamber. DMEM medium containing 10% FBS was added to the lower chamber and the system was incubated for 24 h. Then the upper chamber with invaded cells was fixed with 4% paraformaldehyde and stained with crystal violet for 20 min. Three randomly selected areas were photographed and the glioma cells were counted.

### 2.7. RNA Sequencing and Screening of Differentially Expressed Genes

RNA libraries were prepared from U87 cells treated with vehicle and SU4312 for 24 h. RNAs were sequenced using the BGISEQ-500 platform (BGI Genomics, China), followed by screening differentially expressed genes (DEGs) using the NOISeq method. DEGs were analyzed using Dr. Tom online software (BGI) based on the Kyoto Encyclopedia of Genes and Genomes (KEGG) pathway, with Q-values < 0.05 for the pathways shown in the graph.

### 2.8. Western Blot

U251 and GBM1 cells were treated with SU4312 (10 μM or 20 μM) for 24 h. After extraction, the protein concentration was determined using the Bradford method. Fifty micrograms of sample protein were added to a 10% polyacrylamide gel for electrophoresis and were electrotransferred to polyvinylidene difluoride membranes. The membrane was blocked with 5% skimmed milk or 3% bovine serum albumin for 2 h at room temperature, and the primary antibodies (anti-YAP, anti-AXL, anti-CRY61, anti-GAPDH) were incubated overnight at 4 °C, followed by incubation with the secondary antibody for 2 h at room temperature. The signal was detected by the ECL luminescence system.

### 2.9. Intracranial Cell-Derived Xenograft Models

Mice were purchased from the Laboratory Animal Center of Xuzhou Medical University. The mice were housed under standard conditions at the animal care facility in the Center of Experimental Animals of Institute of Nervous System Diseases, Xuzhou Medical University. All in vivo experiments conducted in this study were approved by The Ethics Committee of Xuzhou Medical University(202104A456). In the YAP-restoring experiment, GFP-luciferase-YAP or GFP-luciferase-vector U87 cells (5 × 10^5^) were intracranially injected into BALB/c male nude mice (male, 5 weeks, 20 g, *n* = 8). After transplantation for 2 days, mice were intragastricly (ig) treated with SU4312 (1 mg/kg, 5 days on, 2 days off) for 3 weeks. In SU4312 and TMZ synergy experiments, GFP-luciferase-U87 cells (5 × 10^5^) or mCherry-luciferase-GL261 cells (2 × 10^5^) were intracranially injected into BALB/c nude mice (male, 5 weeks, 20 g, *n* = 8) or C57BL/6 mice (male, 5 weeks, 20 g, *n* = 8), respectively. After transplantation for 2 days, mice were treated with SU4312 (1 mg/kg, 5 days on, 2 days off, ig) or TMZ alone (7.5 mg/kg, 5 days on, 2 days off, ip). Intracranial tumor growth was monitored using bioluminescence imaging (IVIS Lumina S5, PerkinElmer) at day 7, 14 and 21, respectively. When tumor-bearing mice exhibited signs of depression, hemiparesis and cachexia, they were euthanized with 5% isoflurane anesthesia. The organs (brain, heart, liver, spleen, lungs, kidneys) were removed and fixed in 4% paraformaldehyde for subsequent detection. Tissue sections were stained with H&E staining to observe whether the drug had toxic effects on the major organs of mice. Quantitative analysis of the tumor size was measured using relative fluorescence index. Overall survival was calculated using Kaplan-Meier curves.

### 2.10. RNA Extraction and Quantification Real-Time PCR

Total RNA was extracted from cultured U251, U87 and GBM1 cells or mice xenografts using Trizol (Invitrogen) reagent and was reversely transcribed into first strand cDNA using a reverse transcription kit (Tiangen). PCR was performed using SuperReal PreMix Plus (Tiangen) reagent for real-time fluorescence quantification according to the manufacturer’s instructions. β-actin was used as an internal control gene. The mRNA expression levels of the target genes were calculated using the 2^−ΔΔCt^ method and normalized to that of β-actin. All primer sequences (Table 1) were produced by Sangon Biotech Company.

### 2.11. ELISA Assay

To detect the amount of CCL2, the cell supernatant and tumor homogenate supernatant were collected and determined using a CCL2 ELISA kit (Shanghai Lanpai Company) according to the manufacturer’s instructions.

### 2.12. Immunofluorescence and Immunohistochemistry Staining

The immunohistochemistry (IHC) and immunofluorescence were performed as described previously [36,37]. The cells and brain slices were sequentially incubated with primary and secondary antibodies, and nuclei were counterstained with DAPI or hematoxylin.

### 2.13. Statistical Analysis

The results were representative of experiments expressed as the means ± SD. To analyze significant differences within groups, single comparisons were performed using Student’s *t*-test and multiple comparisons using one-way ANOVA followed by Tukey’s test via GraphPad Prism 8 software. Overall survival curves were calculated by Kaplan–Meier and compared using the log-rank test. *p <* 0.05 were considered statistically significant (* *p <* 0.05, ** *p <* 0.01, *** *p <* 0.001).

## 3. Results

### 3.1. SU4312 Inhibits the Proliferation, Invasion and Migration of Glioma Cells

**Figure 1:**
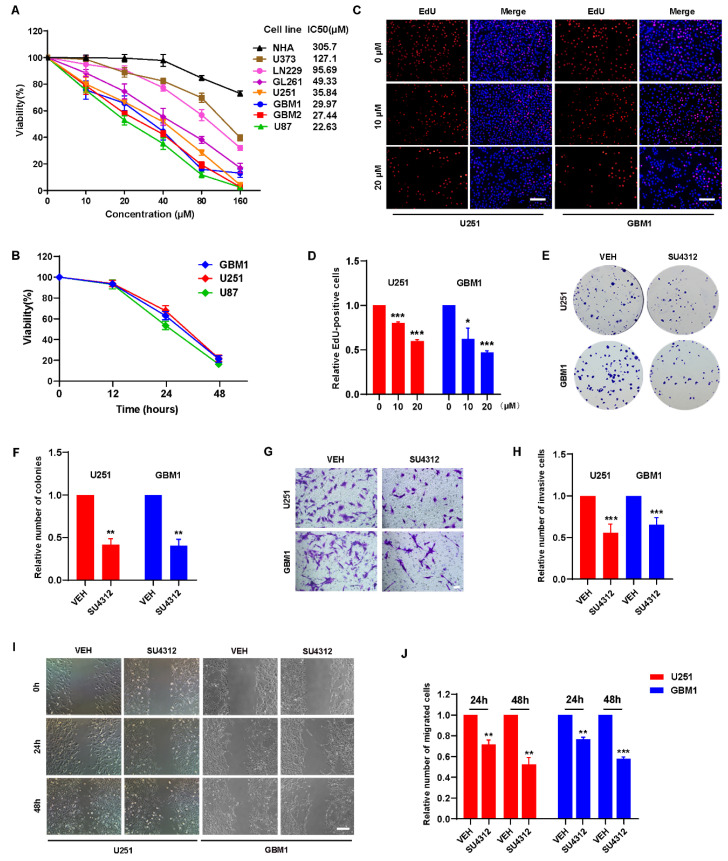
SU4312 inhibits the proliferation, invasion and migration of glioma cells. (**A**). CCK-8 assay of seven glioma cell lines and normal human astrocytes treated with different concentrations of SU4312 for 24 h and the IC50 values of each cell line were calculated. (**B**). CCK-8 assay of three SU4312-sensitive glioma cell lines treated with 20 μM of SU4312 for 0, 12, 24 and 48 h. (**C**,**D**). Representative images (**C**) and quantitative results (**D**) of EdU incorporation assay in U251 and GBM1 cells treated with the indicated concentration of SU4312. Scale bar: 200 μM. (**E**,**F**). Representative images (**E**) and quantitative results (**F**) of colony formation assay after U251 and GBM1 cells treated with SU4312 at 10 μM. (**G**,**H**). Representative images (**G**) and quantitative results (**H**) of invasive cells after U251 and GBM1 cells treated with SU4312 at 10 μM. Scale bar: 50 μM. (**I**,**J**). Representative images (**I**) and quantitative results (**J**) of migratory cells after U251 and GBM1 cells treated with SU4312 for 0, 24 and 48 h at 10 μM. Scale bar: 100 μM. * *p <* 0.05, ** *p <* 0.01, *** *p <* 0.001.

To detect the effect of SU4312 on the viability of glioma cells, the CCK-8 assay was performed in five glioma cell lines (U251, U87, U373, LN229, and GL261), two primary glioma cell lines (GBM1 and GBM2) and human normal astrocytes (NHA). The results showed that SU4312 inhibited the viability of glioma cells in a dose-dependent manner in most of the tested cell lines (Figure 1A), and the half-maximal inhibitory concentration (IC50) values of these cell lines varied from 127.1 μM to 22.63 μM. However, the IC50 value of SU4312 on NHA cells was 305.7 μM, which was 2.4–13.5 times higher than that of glioma cell lines, indicating that SU4312 exhibited high tumor inhibition specificity for glioma cells (Figure 1A). In addition, we selected three glioma cell lines with relatively low IC50 values (U251, U87 and GBM1) for further experiments and found that the inhibitory effect of SU4312 (20 μΜ) on these glioma cells was present in a time-dependent manner (Figure 1B). Moreover, SU4312 significantly reduced EdU positive cells at both 10 μΜ and 20 μΜ (Figure 1C,D). In addition, SU4312 decreased the clone numbers of U251 cells by 58.62% and those of GBM1 cells by 59.65% at 10 μΜ (Figure 1E,F). Next, by using transwell assay, we found that SU4312 reduced the invasive numbers of U251 cells by 44.48% and that of GBM1 cells by 34.80% at 10 μΜ (Figure 1G,H). Furthermore, SU4312 significantly inhibited the migration of glioma cells at 24 h and 48 h under 10 μΜ using a cell migration assay (Figure 1I,J).

### 3.2. SU4312 Inhibits the Transcription and Expression of YAP

**Figure 2:**
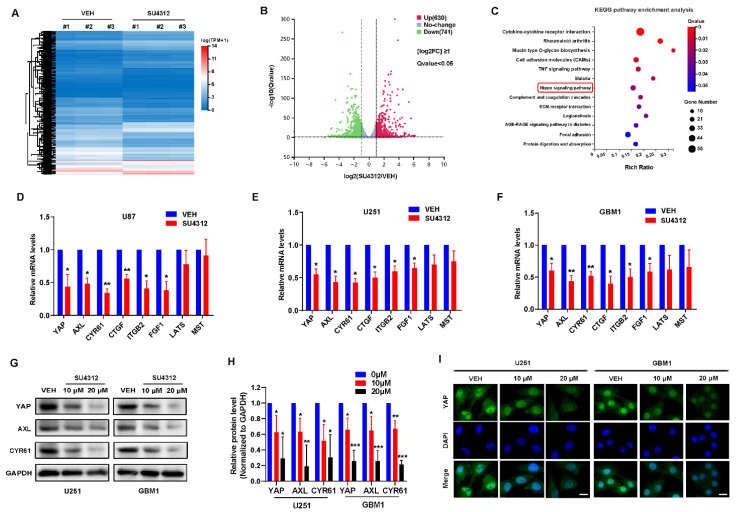
SU4312 inhibits the transcription and expression of YAP. (**A**). Differentially expressed genes (DEGs) in the vehicle and SU4312 treatment groups with triplicates are shown in the heat map. Gradient color barcode shows fold change in gene expression. (**B**). According to the volcano scatter plot of expressed genes, 630 genes were up-regulated and 741 genes were down-regulated after SU4312 treatment. [log2FC] ≥1, Q value < 0.05. (**C**). KEGG pathway enrichment analysis of the DEGs in SU4312 treated cells vs. control cells. (**D**–**F**). qPCR was used to detect the transcription of YAP and the hippo pathway related genes in U87 (**D**), U251 (**E**) and GBM1 cells (**F**). (**G**). Protein expression level of YAP and its target genes were detected after different doses of SU4312 treatment in U251 and GBM1 cells. (**H**). Quantitative analysis of the results in (**G**). (**I**). The expression and localization of YAP (green) after SU4312 treatment were detected by immunofluorescence. Scale bar: 10 μM. * *p <* 0.05, ** *p <* 0.01, *** *p <* 0.001.

To investigate the molecular mechanism of SU4312 inhibiting glioma cell growth, we examined the expression of genes in vehicle- and SU4312-treated cells using high-throughput RNA-sequencing (RNA-seq) analysis (Figure 2A) and screened 1371 DEGs, of which 741 genes were down-regulated and 630 were up-regulated after SU4312 treatment (Figure 2B). The KEGG annotation and pathway enrichment analysis of these DEGs showed that the hippo signal pathway, which we studied for several years, significantly changed after SU4312 treatment (Figure 2C). YAP, the core effector of the hippo signal pathway, was significantly down-regulated. To verify this result, we used qPCR to detect the mRNA level of YAP and its upstream kinases MST and LATS, as well as its target genes AXL, CYR61, CTGF, ITGB2 and FGF1 in U87, U251 and GBM1 cells. The results showed that the mRNA level of MST or LATS showed no change, while those of YAP and its target genes were significantly down-regulated (Figure 2D–F), suggesting that SU4312 inhibits the transcription of YAP. In addition, the protein levels of YAP, AXL and CYR61 significantly decreased in U251 and GBM1 cells after SU4312 treatment (Figure 2G,H). The fluorescence intensity of YAP in U251 and GBM1 cells decreased after SU4312 treatment, while the subcellular location of YAP showed no significant change (Figure 2I). The above results showed that SU4312 treatment inhibits the transcription and expression of YAP. Since YAP is a well-known tumor-promoting gene in gliomas [24,25,26,27,28,29], we speculate that SU4312 may repress the proliferation, invasion and migration of gliomas by inhibiting the transcription and expression of YAP.

### 3.3. The Inhibitory Effect of SU4312 on Glioma Progression Is Partially Restored by YAP Over-Expression

**Figure 3:**
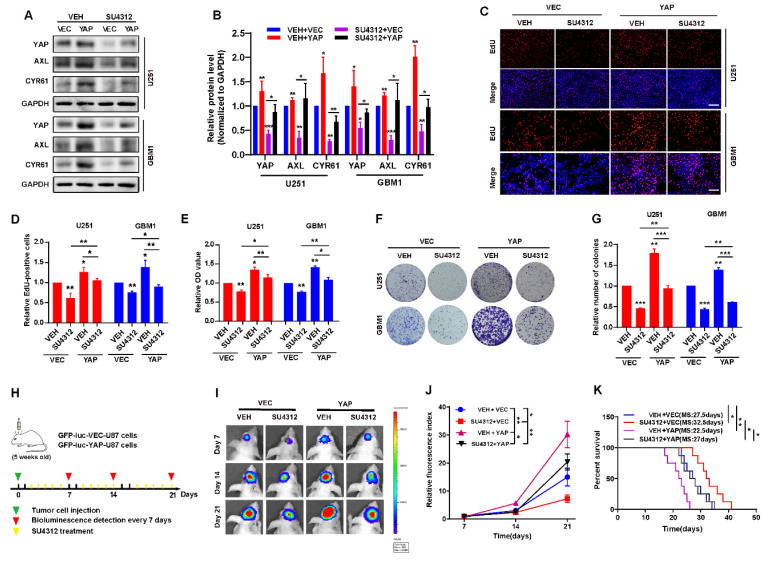
The inhibitory effect of SU4312 on glioma progression is partially restored by YAP over-expression. (**A**). The protein levels of YAP and its downstream molecules (AXL, CYR61) were restored by YAP over-expression after SU4312 (10 μM) treatment. (**B**). Quantitative analysis of the results in (**A**). (**C**,**D**). Representative images (**C**) and quantitative results (**D**) of EdU incorporation assay in U251 and GBM1 cells treated with SU4312 (10 μM) with or without YAP over-expression. Scale bar: 200 μM. (**E**). CCK-8 assay indicates that the inhibition effects of SU4312 (10 μM) were partially restored by YAP over-expression on U251 and GBM1 cells. (**F**,**G**). Representative images (**F**) and quantitative results (**G**) of colony formation assay in U251 and GBM1 cells treated with SU4312(10 μM), with or without YAP over-expression. (**H**). Schematic representation of U87 cell-derived allograft experimental workflow. (**I**). Representative bioluminescence images of intracranial xenografts bearing YAP over-expression or vector U87 cells on the indicated days. (**J**). Quantitative analysis of the tumor size by relative fluorescence index. (**K**). Kaplan-Meier analysis of the median survival time of mice. * *p <* 0.05, ** *p <* 0.01, *** *p <* 0.001.

To demonstrate whether YAP is involved in SU4312′s inhibitory effect on gliomas, we constructed U251 and GBM cell lines that stably over-express wide-type YAP. The results showed that exogenous YAP partially restored the decrease in YAP and its target genes (AXL and CYR61) induced by SU4312 treatment (Figure 3A,B). When examined by EdU incorporation assay, CCK8 assay and colony formation assay, we found that the over-expression of YAP promoted the proliferation of U251 and GBM1 cells, and partially abolished the inhibitory effect of SU4312 on glioma cell growth (Figure 3C–G). Additionally, in order to explore whether YAP is involved in the inhibitory effect of SU4312 on glioma growth in vivo, we constructed an intracranial orthotopic transplanted tumor model (Figure 3H). U87 cells labeled with GFP-luciferase, with or without YAP over-expression, were implanted into the brain of nude mice, and animal in vivo imaging was performed on day 7, 14 and 21. Twenty-one days after implantation, the tumor size of the SU4312 treatment group was smaller, while that of the YAP over-expression group was larger than that of the control group. As expected, the over-expression of YAP partially eliminated the antitumor effect of SU4312 (Figure 3I,J). Meanwhile, Kaplan–Meier analysis showed that the survival time of SU4312 group was longer, while that of the YAP over-expression group was shorter than that of the control group. In addition, the over-expression of YAP blocked the survival benefit of SU4312 on glioma growth (Figure 3K).

### 3.4. Combining SU4312 with TMZ Inhibits Glioma Cell Growth In Vitro

**Figure 4:**
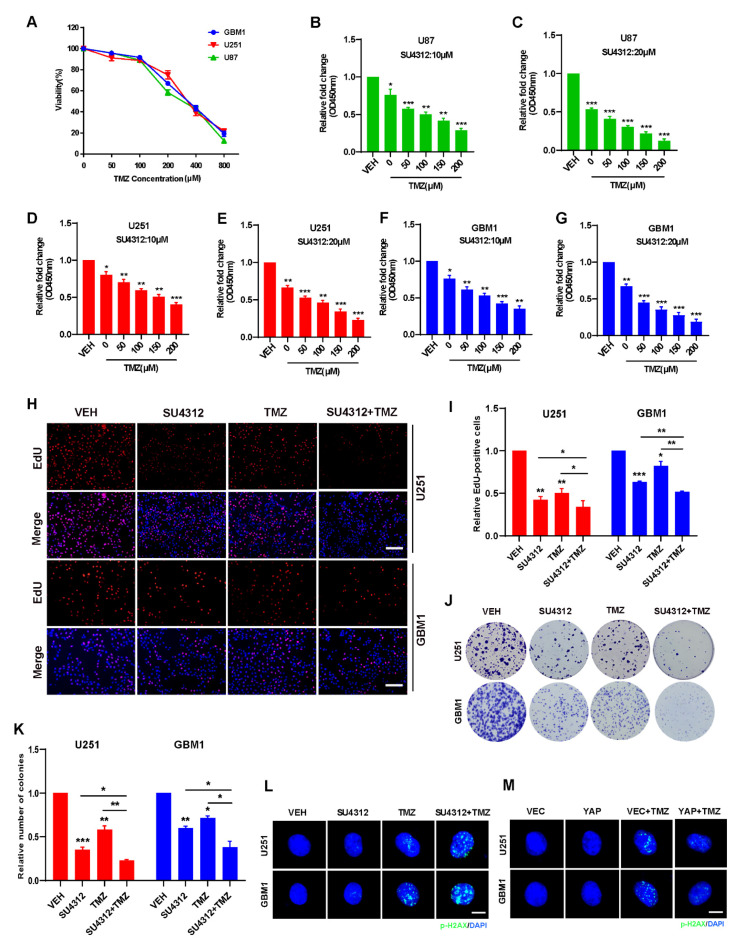
Combining SU4312 with TMZ inhibits glioma cell growth in vitro. (**A**). CCK-8 assay was used to detect the cell viability of three glioma cells treated with TMZ at different concentrations for 24 h. (**B**–**G**). Cell viability of U87 (B&C), U251 (D&E) and GBM1 (F&G) cells after SU4123 (10 μM or 20 μM) treatment was concomitant with increased TMZ concentrations (0, 50, 100, 150, 200 μM). (**H**,**I**). Representative images (**H**) and quantitative results (**I**) of EdU incorporation assay in U251 and GBM1 cells treated with SU4312 (20 μM) and TMZ (300 μM). Scale bar: 200 μM. (**J**,**K**). Representative images (**J**) and quantitative results (**K**) of colony formation assay in U251 and GBM1 cells treated with SU4312 (10 μM) and TMZ (300 μM). (**L**). The p-H_2_AX expression levels of U251 and GBM1 cells treated with SU4312 (10 μM) and TMZ (300 μM) was detected by immunofluorescence. (**M**). Immunofluorescence was used to detect the expression of p-H_2_AX in U251 and GBM1 cells after TMZ treatment (300 μM) with or without YAP over-expression. Scale bar: 5 μM. * *p <* 0.05, ** *p <* 0.01, *** *p <* 0.001.

TMZ is the first-line drug for effective treatment of gliomas [31,40]. Since SU4312 can effectively cross the blood–brain barrier and has an obvious inhibitory effect on gliomas, we combined SU4312 with TMZ to observe whether they had a synergistic effect. First, we detected the cell viability of U87, U251 and GBM1 cells after different concentrations of TMZ treatment (Figure 4A) and found that the IC50 was 288.9 μM for U87 cells, 342 μM for U251 cells, and 333.6 μM for GBM1 cells, respectively. Then, we examined the cell viability by combining SU4312 (10 μΜ or 20 μΜ) with a gradually increasing TMZ concentration ranging from 0 to 200 μM (Figure 4B–G). As shown in Figure 4B–G, in three cell lines, the cell proliferation rate decreased after SU4312 treatment and was further inhibited when treatment was combined with different concentrations of TMZ. In addition, we calculated the combination index (CI) value of SU4312 and TMZ according to Compusyn [36,41] and found that SU4312 had a synergistic effect with TMZ in all three glioma cells for all CI values less than 1 (Table 2). Furthermore, when SU4312 (20 μΜ) was combined with TMZ (300 μΜ), either the number of EdU-positive cells or the colonies of the combination group were significantly lower than those of any single drug group (Figure 4H–K), suggesting that SU4312 and TMZ had a synergistic anti-proliferative effect on glioma in vitro. Moreover, p-H2AX immunofluorescence was used to detect therapy-induced DNA damage [42]. The results show that TMZ-induced DNA damage was enhanced by SU4312 treatment (Figure 4L). In contrast, TMZ-induced DNA damage decreased after the over-expression of YAP (Figure 4M). Hence, we speculate that SU4312 may have a synergistic effect with TMZ on glioma cell growth by down-regulating YAP and consequently increasing DNA damage.

### 3.5. Combining SU4312 with TMZ Inhibits Glioma Progression In Vivo

**Figure 5:**
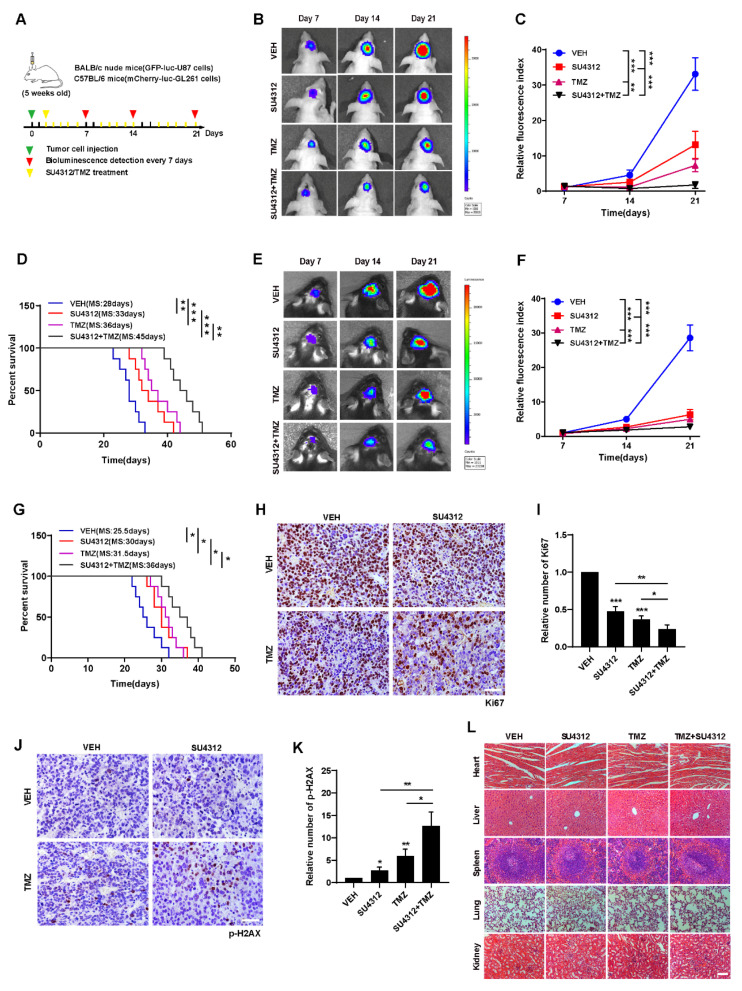
Combining SU4312 with TMZ inhibits glioma progression in vivo. (**A**). Schematic representation of cell-derived allograft experimental workflow. (**B**). Representative bioluminescence images of intracranial xenografts bearing GFP-luc-U87 cells after SU4312 with or without TMZ treatment on the indicated days in immunodeficient mice (BALB/c nude mice). (**C**). Quantitative analysis of the tumor size by relative fluorescence index. (**D**). Kaplan–Meier analysis of the median survival time of immunodeficient mice. (**E**). Representative bioluminescence images of intracranial xenografts bearing mCherry-luc-GL261 cells after SU4312 with or without TMZ treatment on the indicated days in immunocompetent mice (C57BL/6). (**F**). Quantitative analysis of the tumor size by relative fluorescence index. (**G**). Kaplan–Meier analysis of the median survival time of immunocompetent mice. (**H**–**K**). Immunohistochemical detection of representative images (**H**,**J**) and statistical results (**I**,**K**) of the number of Ki67 and p-H_2_AX positive cells in tumor tissues of mice. Scale bar: 50 μM. (**L**). H&E staining was used to detect the histopathological changes of the heart, liver, spleen, lung and kidney of mice treated with SU4312 alone or combined with TMZ. Scale bar: 100 μM. * *p <* 0.05, ** *p <* 0.01, *** *p <* 0.001.

To evaluate the therapeutic efficacy of SU4312 combined with TMZ in vivo, we constructed intracranial orthotopic tumor models in immunodeficient (BALB/c nude mice) and immunocompromised mice (C57BL/6 mice), respectively (Figure 5A). As shown in Figure 5B,E and Figure 5C,F, the tumor size of the SU4312 and TMZ combination group was significantly smaller than those of monotherapy after 21 days of implantation, and the survival time of the SU4312 and TMZ combination group was significantly longer than those of monotherapy (Figure 5D,G). In addition, the number of Ki67 positive cells in the SU4312 combined with TMZ group was lower than those in the single drug treatment group, which further confirmed the synergistic effect of SU4312 and TMZ (Figure 5H,I). Meanwhile, the expression of p-H2AX in the combination group was higher than those of the TMZ or SU4312 monotherapy group, which also suggests that SU4312 could enhance the DNA damage induced by TMZ treatment in vivo (Figure 5J,K). Finally, using H&E staining, we did not observe any histopathological changes in the heart, liver, spleen, lung and kidney after SU4312 with or without TMZ treatment (Figure 5L). Taken together, the above results indicate that SU4312 may be a safe and effective drug for the treatment of glioma, and a potential sensitizer for TMZ both in vitro and in vivo.

### 3.6. SU4312 Inhibits CCL2 Expression by Down-Regulating YAP, Thereby Improving Anti-tumor Immune Microenvironment

**Figure 6:**
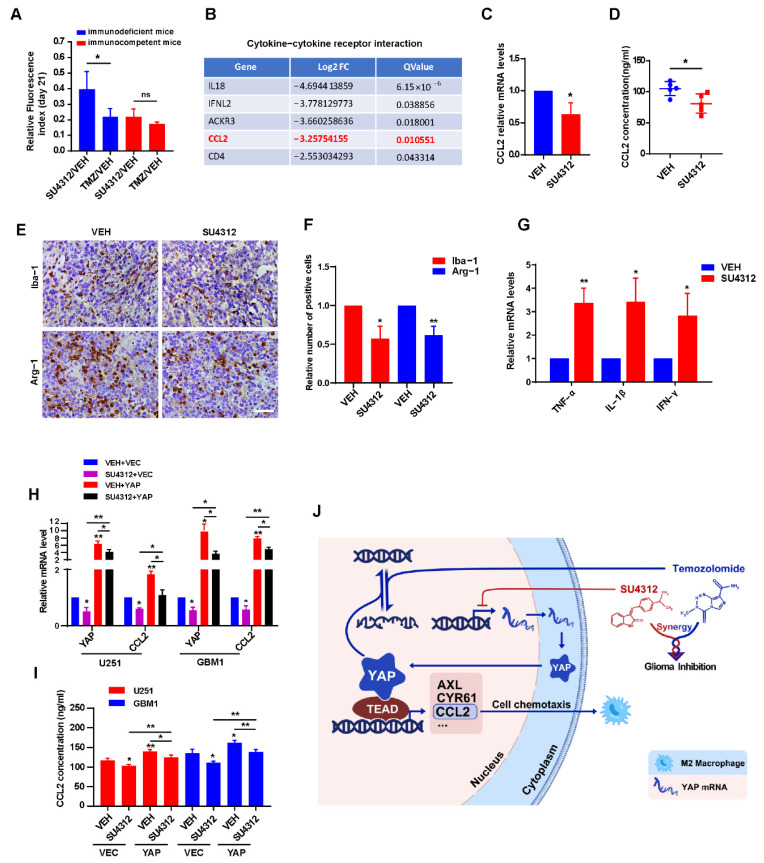
SU4312 inhibits CCL2 expression by down-regulating YAP, thereby improving anti-tumor immune microenvironment. (**A**). Quantitative analysis of the tumor size by relative fluorescence index after SU4312 treatment at 21 days after implantation between immunodeficient mice and immunocompetent mice. (**B**). In the “cytokine–cytokine receptor interaction” module, CCL2 was in the top five down-regulated genes after SU4312 treatment. (**C**,**D**). The mRNA and protein levels of CCL2 in tumor tissues in SU4312 and control group were detected by qPCR (**C**) and ELISA (**D**). (**E**,**F**). Representative images (**E**) and statistical results (**F**) show the changes in the proportion of Iba-1 and Arg-1 positive cells in SU4312 and control group detected by immunohistochemistry. Scale bar: 50 μM. (**G**). The immune-promoting cytokines (TNF-α, IL-1β and IFN-γ) in tumor tissues in SU4312 and control group were detected by qPCR. (**H**,**I**). The mRNA and protein levels of CCL2 in U251 and GBM1 cells treated with SU4312 with or without YAP over-expression were detected by qPCR (**H**) and ELISA (**I**). (**J**). Working model: SU4312 inhibits glioma progression by down-regulating YAP, sensitizes the antitumor effect of TMZ and promotes the formation of anti-tumor immune microenvironment. * *p <* 0.05, ** *p <* 0.01.

In the above in vivo experiments, we unexpectedly observed that the anti-tumor effect of SU4312 in immunodeficient mice was weaker than that in immunocompetent mice compared to the effect of TMZ (Figure 6A), indicating that the immune system is involved in the inhibitory effect of SU4312 on glioma growth. By analyzing the RNA-seq results, we found that the “cytokine–cytokine receptor interaction” module ranked first in KEGG annotation and pathway enrichment analysis (Figure 2C). Therefore, we speculated that SU4312 may promote immunity and then inhibit glioma progression. In the “cytokine–cytokine receptor interaction” module, we surprisingly found that CCL2, which has been reported to be a target gene of YAP in recent studies [43,44], was in the top five down-regulated genes after SU4312 treatment (Figure 6B). As a well-known molecule, CCL2 could recruit M2 macrophages to promote tumor progression [45,46,47], and its reduction means that the tumor immune microenvironment was predisposed to anti-tumor [46,47]. Therefore, we took the tumor tissues of mice in the control and SU4312 treatment group for qPCR (Figure 6C) and ELISA (Figure 6D), and found that the expression of CCL2 significantly decreased in tumor tissues with SU4312 treatment. In addition, the number of macrophages (Iba-1 positive cells) and M2 macrophages (Arg-1 positive cells) in the SU4312 treatment group was significantly lower than those in the control group by immunohistochemistry (Figure 6E,F). Furthermore, when examined by qPCR, we found that the expression of immune-promoting cytokines (TNF-α, IL-1β and IFN-γ) in SU4312-treated tumor tissues was significantly higher than that in the control group (Figure 6G). These results hint that SU4312 may improve the immune-promoting microenvironment. In addition, as it is reported that YAP positively regulates CCL2 [43,44], we further used U251 and GBM1 cells stably over-expressing YAP to detect the changes in CCL2 by qPCR and ELISA. As shown in Figure 6H,I, the over-expression of YAP increased the expression of CCL2, while SU4312 treatment decreased the expression. Moreover, the SU4312-induced CCL2 decrease was eliminated by YAP over-expression. Overall, SU4312 may inhibit CCL2 expression by down-regulating YAP, thereby promoting anti-tumor immunity and inhibiting glioma progression.

## 4. Discussion

Glioblastoma is one of the most malignant glioma types with rapid progression and poor treatment effects [3,4]. Chemotherapy has shown certain efficacy in the treatment of GBM, but the penetration of the blood–brain barrier, drug resistance and other factors limit its wide application [48,49]. The receptor tyrosine kinase inhibitor SU4312 is reported to inhibit tumor angiogenesis [5,6,7,8]. In this study, we identified that SU4312 exhibits an anti-glioma effect by down-regulating YAP and CCL2, improving the tumor immune microenvironment. In addition, SU4312 may serve as a potential sensitizer for TMZ by inducing DNA damage (Figure 6J).

The hippo pathway plays an important role in the occurrence and development of tumors [15,29]. YAP, as the core element of the hippo pathway, has been proven to be an oncogene and to promote tumor progression [21,50]. Our results show that YAP and its target genes significantly decreased after SU4312 treatment, while the upstream molecules MST and LATS remained unchanged. Previous studies in our laboratory clarified that down-regulation of YAP inhibited glioma progression, while over-expression of YAP has the opposite effect [25,26,37]. As a transcriptional coactivator, entry into the nucleus of YAP is necessary to exert its function [20,36]. However, in contrast to imipramine, which inhibits YAP by down-regulating its expression and decreasing its nuclear translocation [36], SU4312 only down-regulated the expression of YAP without affecting its nuclear localization. Therefore, SU4312 may inhibit YAP only by decreasing the transcription of YAP. How SU4312 down-regulates YAP transcription deserves further study.

TMZ is a first-line drug for the treatment of glioma, especially for GBM. TMZ treatment efficacy differs among patients [40,49] and TMZ-acquired resistance has been shown [33]. Therefore, new strategies to improve TMZ efficacy are being explored [48,49]. In the present study, we clarified that SU4312 sensitized the anti-tumor effect of TMZ in vitro and in vivo by enhancing the DNA damage induced by TMZ. In a previous study, we elucidated the mechanism by which YAP resists DNA damage and promotes its repair [37]. Since SU4312 can down-regulate the expression of YAP, we speculate that SU4312 may enhance DNA damage by down-regulating YAP, thereby sensitizing TMZ. SU4312 may be a new sensitizer for TMZ, but the detailed mechanism of its synergistic effect needs further elucidation.

In recent years, immunotherapy has become increasingly important in the treatment of gliomas [51,52]. The immune microenvironment plays an important role in the occurrence and development of gliomas [52,53]. It has been reported that some chemotherapeutic drugs can shift the glioma-immune microenvironment towards tumor suppression [54,55]. Our results unexpectedly found that macrophages (especially M2 macrophages) decreased and immune-promoting cytokines increased after SU4312 treatment, suggesting that SU4312 can reprogram the anti-tumor immune microenvironment of gliomas. CCL2 was significantly down-regulated after SU4312 treatment, and the number of M2 macrophages was subsequently decreased, indicating that the effect of SU4312 on the glioma-immune microenvironment is mediated by the down-regulation of CCL2. Moreover, YAP has been reported to regulate CCL2 in tumors and CCL2 may be a target gene of YAP [43,44]. Our results also support this notion in gliomas. Together, the above results show that SU4312 down-inhibits CCL2 expression by inhibiting YAP, thereby down-regulating anti-tumor immunity and inhibiting glioma progression.

## 5. Conclusions

In summary, the anti-tumor effect of SU4312 was demonstrated in glioma for the first time. Our findings suggest that SU4312 inhibits glioma progression by down-regulating the transcription and expression of YAP. Furthermore, SU4312 effectively synergizes the anti-tumor effect of TMZ and may act as a sensitizer for TMZ. SU4312 also improves the anti-tumor immune microenvironment, which presumably results from the down-regulation of CCL2 due to the inhibition of YAP. SU4312, as an anti-tumor drug with significant efficacy, may provide a possible new treatment approach for patients with gliomas.

## Figures and Tables

**Table 1:** Primer sequences used in this study.

Gene Name	Primer Sequences (Forward: 5′—3′)	Primer Sequences (Reverse: 5′—3′)
YAP (human)	CACAGCTCAGCATCTTCGAC	TATTCTGCTGCACTGGTGGA
AXL (human)	AAGAGCGATGTGTGGTCCTT	CGATTTCCCTGGCGCAGATA
CYR61 (human)	CCTTGTGGACAGCCAGTGTA	ACTTGGGCCGGTATTTCTTC
CTGF (human)	AGGAGTGGGTGTGTGACGA	CCAGGCAGTTGGCTCTAATC
ITGB2 (human)	CAGGGCAGACTGGTAGCAAA	CACTCCTGAGAGAGGACGCA
FGF1 (human)	GTGGATGGGACAAGGGACAG	ATTTGGTGTCTGTGAGCCGT
LATS (human)	ACTCACAGACAGATGTAGGA	GAGAGGTGGTGGAGGATAGC
MST (human)	ACAAATCCTCCTCCCACATTCCG	CACTCCTGACAAATGGGTGCTG
CCL2 (human)	ACCAGCAGCAAGTGTCCCAAAG	TTTGCTTGTCCAGGTGGTCCATG
CCL2 (mouse)	TTTTTGTCACCAAGCTCAAGAG	TTCTGATCTCATTTGGTTCCGA
IFN-γ (mouse)	CTTGAAAGACAATCAGGCCATC	CTTGGCAATACTCATGAATGCA
TNF-α (mouse)	TTGCTCTGTGAAGGGAATGG	GGCTCTGAGGAGTAGACAATAAAG
IL-1β (mouse)	ATGGGCAACCACTTACCTATTT	GTTCTAGAGAGTGCTGCCTAATG
β-actin (human)	CCAACCGCGAGAAGATGA	CCAGAGGCGTACAGGGATAG
β-actin (mouse)	CTACCTCATGAAGATCCTGACC	CACAGCTTCTCTTTGATGTCAC

**Table 2:** Combination index (CI) of SU4312 and TMZ in U87, U251 and GBM1 cells.

SU4312 (μM)	TMZ (μM)	U87		U251		GBM1	
		Inhibition Ratio	CI Value	Inhibition Ratio	CI Value	Inhibition Ratio	CI Value
10	50	0.427	0.743	0.300	0.823	0.389	0.698
10	100	0.500	0.800	0.408	0.788	0.469	0.700
10	150	0.587	0.796	0.495	0.767	0.577	0.635
10	200	0.714	0.683	0.600	0.682	0.650	0.615
20	50	0.591	0.873	0.472	0.845	0.550	0.719
20	100	0.697	0.775	0.537	0.843	0.641	0.637
20	150	0.780	0.690	0.658	0.691	0.718	0.573
20	200	0.880	0.512	0.770	0.536	0.806	0.466

CI value < 1 indicates that there is a synergistic effect of SU4312 combined with TMZ.

## Data Availability

The data used to support the findings of this study are available from the corresponding author upon request.

## References

[1] Yang P., Wang Y., Peng X., You G., Zhang W., Yan W., Bao Z., Wang Y., Qiu X., Jiang T. (2013). Management and survival rates in patients with glioma in China (2004-2010): A retrospective study from a single-institution. J. Neuro-Oncol..

[2] Jiang T., Mao Y., Ma W., Mao Q., You Y., Yang X., Jiang C., Kang C., Li X., Chen L. (2016). CGCG clinical practice guidelines for the management of adult diffuse gliomas. Cancer Lett..

[3] Bush N.A., Chang S.M., Berger M.S. (2017). Current and future strategies for treatment of glioma. Neurosurg. Rev..

[4] Jiang T., Nam D.H., Ram Z., Poon W.S., Wang J., Boldbaatar D., Mao Y., Ma W., Mao Q., You Y. (2021). Clinical practice guidelines for the management of adult diffuse gliomas. Cancer Lett..

[5] Abbaspour Babaei M., Kamalidehghan B., Saleem M., Huri H.Z., Ahmadipour F. (2016). Receptor tyrosine kinase (c-Kit) inhibitors: A potential therapeutic target in cancer cells. Drug Des. Dev. Ther..

[6] Du Z., Lovly C.M. (2018). Mechanisms of receptor tyrosine kinase activation in cancer. Mol. Cancer.

[7] Yamaoka T., Kusumoto S., Ando K., Ohba M., Ohmori T. (2018). Receptor Tyrosine Kinase-Targeted Cancer Therapy. Int. J. Mol. Sci..

[8] Esteban-Villarrubia J., Soto-Castillo J.J., Pozas J., San Roman-Gil M., Orejana-Martin I., Torres-Jimenez J., Carrato A., Alonso-Gordoa T., Molina-Cerrillo J. (2020). Tyrosine Kinase Receptors in Oncology. Int. J. Mol. Sci..

[9] Sun L., Tran N., Tang F., App H., Hirth P., McMahon G. (1998). Synthesis and Biological Evaluations of 3-Substituted Indolin-2-ones: A Novel Class of Tyrosine Kinase Inhibitors That Exhibit Selectivity toward Particular Receptor Tyrosine Kinases. J. Med. Chem..

[10] Lee J.S., Kang Y., Kim J.T., Thapa D., Lee E.S., Kim J.A. (2012). The anti-angiogenic and anti-tumor activity of synthetic phenylpropenone derivatives is mediated through the inhibition of receptor tyrosine kinases. Eur. J. Pharmacol..

[11] Schultheiss C., Blechert B., Gaertner F.C., Drecoll E., Mueller J., Weber G.F., Drzezga A., Essler M. (2006). In vivo characterization of endothelial cell activation in a transgenic mouse model of Alzheimer’s disease. Angiogenesis.

[12] Guo B., Hu S., Zheng C., Wang H., Luo F., Li H., Cui W., Yang X., Cui G., Mak S. (2017). Substantial protection against MPTP-associated Parkinson’s neurotoxicity in vitro and in vivo by anti-cancer agent SU4312 via activation of MEF2D and inhibition of MAO-B. Neuropharmacology.

[13] Cui W., Zhang Z., Li W., Hu S., Mak S., Zhang H., Han R., Yuan S., Li S., Sa F. (2013). The anti-cancer agent SU4312 unexpectedly protects against MPP(+) -induced neurotoxicity via selective and direct inhibition of neuronal NOS. Br. J. Pharmacol..

[14] McMillin D.W., Delmore J., Weisberg E., Negri J.M., Geer D.C., Klippel S., Mitsiades N., Schlossman R.L., Munshi N.C., Kung A.L. (2010). Tumor cell-specific bioluminescence platform to identify stroma-induced changes to anticancer drug activity. Nat. Med..

[15] Yu F.X., Zhao B., Guan K.L. (2015). Hippo Pathway in Organ Size Control, Tissue Homeostasis, and Cancer. Cell.

[16] Zheng Y., Pan D. (2019). The Hippo Signaling Pathway in Development and Disease. Dev. Cell.

[17] Nguyen C.D.K., Yi C. (2019). YAP/TAZ Signaling and Resistance to Cancer Therapy. Trends. Cancer.

[18] Zhang Y., Wang X., Zhou X. (2022). Functions of Yes-association protein (YAP) in cancer progression and anticancer therapy resistance. Brain Sci. Adv..

[19] Orr B.A., Bai H., Odia Y., Jain D., Anders R.A., Eberhart C.G. (2011). Yes-associated protein 1 is widely expressed in human brain tumors and promotes glioblastoma growth. J. Neuropath. Exp. Neur..

[20] Ouyang T., Meng W., Li M., Hong T., Zhang N. (2020). Recent Advances of the Hippo/YAP Signaling Pathway in Brain Development and Glioma. Cell Mol. Neurobiol.

[21] Avruch J., Zhou D., Bardeesy N. (2012). YAP oncogene overexpression supercharges colon cancer proliferation. Cell Cycle.

[22] Jang M., An J., Oh S.W., Lim J.Y., Kim J., Choi J.K., Cheong J.H., Kim P. (2021). Matrix stiffness epigenetically regulates the oncogenic activation of the Yes-associated protein in gastric cancer. Nat. Biomed. Eng..

[23] Wang J., Ma L., Weng W., Qiao Y., Zhang Y., He J., Wang H., Xiao W., Li L., Chu Q. (2013). Mutual interaction between YAP and CREB promotes tumorigenesis in liver cancer. Hepatology.

[24] Zhang H., Geng D., Gao J., Qi Y., Shi Y., Wang Y., Jiang Y., Zhang Y., Fu J., Dong Y. (2016). Expression and significance of Hippo/YAP signaling in glioma progression. Tumour Biol..

[25] Wang Y., Pan P., Wang Z., Zhang Y., Xie P., Geng D., Jiang Y., Yu R., Zhou X. (2017). beta-catenin-mediated YAP signaling promotes human glioma growth. J. Exp. Clin. Cancer Res..

[26] Zhang Y., Xie P., Wang X., Pan P., Wang Y., Zhang H., Dong Y., Shi Y., Jiang Y., Yu R. (2018). YAP Promotes Migration and Invasion of Human Glioma Cells. J. Mol. Neurosci..

[27] Zhao M., Zhang Y., Jiang Y., Wang K., Wang X., Zhou D., Wang Y., Yu R., Zhou X. (2021). YAP promotes autophagy and progression of gliomas via upregulating HMGB1. J. Exp. Clin. Cancer Res..

[28] Vigneswaran K., Boyd N.H., Oh S.Y., Lallani S., Boucher A., Neill S.G., Olson J.J., Read R.D. (2021). YAP/TAZ Transcriptional Coactivators Create Therapeutic Vulnerability to Verteporfin in EGFR-mutant Glioblastoma. Clin. Cancer Res..

[29] Masliantsev K., Karayan-Tapon L., Guichet P.O. (2021). Hippo Signaling Pathway in Gliomas. Cells.

[30] Oku Y., Nishiya N., Shito T., Yamamoto R., Yamamoto Y., Oyama C., Uehara Y. (2015). Small molecules inhibiting the nuclear localization of YAP/TAZ for chemotherapeutics and chemosensitizers against breast cancers. FEBS Open Bio.

[31] Carter T.C., Medina-Flores R., Lawler B.E. (2018). Glioblastoma Treatment with Temozolomide and Bevacizumab and Overall Survival in a Rural Tertiary Healthcare Practice. BioMed Res. Int..

[32] Stupp R., Mason W.P., van den Bent M.J., Weller M., Fisher B., Taphoorn M.J., Belanger K., Brandes A.A., Marosi C., Bogdahn U. (2005). Radiotherapy plus concomitant and adjuvant temozolomide for glioblastoma. N. Engl. J. Med..

[33] Lee S.Y. (2016). Temozolomide resistance in glioblastoma multiforme. Genes Dis..

[34] Wang C., Jiang M., Hou H., Lin Q., Yan Z., Zhang X. (2018). Apatinib suppresses cell growth and metastasis and promotes antitumor activity of temozolomide in glioma. Oncol. Lett..

[35] Han S., Kim S., Chen Z., Shin H.K., Lee S.Y., Moon H.E., Paek S.H., Park S. (2020). 3D Bioprinted Vascularized Tumour for Drug Testing. Int. J. Mol. Sci..

[36] Wang Y., Wang X., Wang X., Wu D., Qi J., Zhang Y., Wang K., Zhou D., Meng Q.M., Nie E. (2021). Imipramine impedes glioma progression by inhibiting YAP as a Hippo pathway independent manner and synergizes with temozolomide. J. Cell Mol. Med..

[37] Zhang Y., Wang Y., Zhou D., Wang K., Wang X., Wang X., Jiang Y., Zhao M., Yu R., Zhou X. (2021). Radiation-induced YAP activation confers glioma radioresistance via promoting FGF2 transcription and DNA damage repair. Oncogene.

[38] Wang X., Wang Z., Zhang Y., Wang Y., Zhang H., Xie S., Xie P., Yu R., Zhou X. (2019). Golgi phosphoprotein 3 sensitizes the tumour suppression effect of gefitinib on gliomas. Cell Prolif..

[39] Jiao S., Wang H., Shi Z., Dong A., Zhang W., Song X., He F., Wang Y., Zhang Z., Wang W. (2014). A peptide mimicking VGLL4 function acts as a YAP antagonist therapy against gastric cancer. Cancer Cell.

[40] Chua J., Nafziger E., Leung D. (2019). Evidence-Based Practice: Temozolomide Beyond Glioblastoma. Curr. Oncol. Rep..

[41] Nagaraja S., Vitanza N.A., Woo P.J., Taylor K.R., Liu F., Zhang L., Li M., Meng W., Ponnuswami A., Sun W. (2017). Transcriptional Dependencies in Diffuse Intrinsic Pontine Glioma. Cancer Cell.

[42] Yi G.Z., Huang G., Guo M., Zhang X., Wang H., Deng S., Li Y., Xiang W., Chen Z., Pan J. (2019). Acquired temozolomide resistance in MGMT-deficient glioblastoma cells is associated with regulation of DNA repair by DHC2. Brain J. Neurol..

[43] Xu S., Koroleva M., Yin M., Jin Z.G. (2016). Atheroprotective laminar flow inhibits Hippo pathway effector YAP in endothelial cells. Transl. Res..

[44] Cao M.X., Zhang W.L., Yu X.H., Wu J.S., Qiao X.W., Huang M.C., Wang K., Wu J.B., Tang Y.J., Jiang J. (2020). Interplay between cancer cells and M2 macrophages is necessary for miR-550a-3-5p down-regulation-mediated HPV-positive OSCC progression. J. Exp. Clin. Cancer Res. CR.

[45] Miyamoto T., Murakami R., Hamanishi J., Tanigaki K., Hosoe Y., Mise N., Takamatsu S., Mise Y., Ukita M., Taki M. (2022). B7-H3 Suppresses Antitumor Immunity via the CCL2-CCR2-M2 Macrophage Axis and Contributes to Ovarian Cancer Progression. Cancer Immunol. Res..

[46] Li X., Yao W., Yuan Y., Chen P., Li B., Li J., Chu R., Song H., Xie D., Jiang X. (2017). Targeting of tumour-infiltrating macrophages via CCL2/CCR2 signalling as a therapeutic strategy against hepatocellular carcinoma. Gut.

[47] Yang H., Zhang Q., Xu M., Wang L., Chen X., Feng Y., Li Y., Zhang X., Cui W., Jia X. (2020). CCL2-CCR2 axis recruits tumor associated macrophages to induce immune evasion through PD-1 signaling in esophageal carcinogenesis. Mol. Cancer.

[48] Da Ros M., De Gregorio V., Iorio A.L., Giunti L., Guidi M., de Martino M., Genitori L., Sardi I. (2018). Glioblastoma Chemoresistance: The Double Play by Microenvironment and Blood-Brain Barrier. Int. J. Mol. Sci..

[49] Ou A., Yung W.K.A., Majd N. (2020). Molecular Mechanisms of Treatment Resistance in Glioblastoma. Int. J. Mol. Sci..

[50] Thompson B.J. (2020). YAP/TAZ: Drivers of Tumor Growth, Metastasis, and Resistance to Therapy. Bioessays.

[51] Schumacher T., Bunse L., Pusch S., Sahm F., Wiestler B., Quandt J., Menn O., Osswald M., Oezen I., Ott M. (2014). A vaccine targeting mutant IDH1 induces antitumour immunity. Nature.

[52] Sokratous G., Polyzoidis S., Ashkan K. (2017). Immune infiltration of tumor microenvironment following immunotherapy for glioblastoma multiforme. Hum. Vaccin. Immunother..

[53] Pinton L., Masetto E., Vettore M., Solito S., Magri S., D’Andolfi M., Del Bianco P., Lollo G., Benoit J.P., Okada H. (2019). The immune suppressive microenvironment of human gliomas depends on the accumulation of bone marrow-derived macrophages in the center of the lesion. J. Immunother. Cancer.

[54] von Roemeling C.A., Wang Y., Qie Y., Yuan H., Zhao H., Liu X., Yang Z., Yang M., Deng W., Bruno K.A. (2020). Therapeutic modulation of phagocytosis in glioblastoma can activate both innate and adaptive antitumour immunity. Nat. Commun..

[55] Zhang X.N., Yang K.D., Chen C., He Z.C., Wang Q.H., Feng H., Lv S.Q., Wang Y., Mao M., Liu Q. (2021). Pericytes augment glioblastoma cell resistance to temozolomide through CCL5-CCR5 paracrine signaling. Cell Res..

